# Differential expression profiles of long noncoding RNAs and mRNAs in human bone marrow mesenchymal stem cells after exposure to a high dosage of dexamethasone

**DOI:** 10.1186/s13287-020-02040-8

**Published:** 2021-01-06

**Authors:** Tao Li, Yingxing Xu, Yingzhen Wang, Yaping Jiang

**Affiliations:** 1 Department of Joint Surgery, The Affiliated Hospital of Qingdao University, Qingdao, 266003 China; 2 Qingdao University, Qingdao, 266071 Shandong China; 3 Medical Department of Qingdao University, Qingdao, 266071 Shandong China; 4 Department of Oral Implantology, The Affiliated Hospital of Qingdao University, Qingdao, 266003 China

**Keywords:** MSCs, Gene expression, Long noncoding RNA, Apoptosis, Dexamethasone

## Abstract

**Background:**

Abnormalities in apoptosis, cell cycle, proliferation, and differentiation of human bone marrow mesenchymal stem cells (hBMSCs) significantly impact bone metabolism and remodeling, resulting in various skeletal disorders. Long-term exposure to a high dosage of dexamethasone (Dex) induces apoptosis and inhibits the proliferation of mesenchymal stromal cells (MSCs), which are probable primary causes of various skeletal disorders. However, to date, the exact mechanisms of action of Dex on hBMSCs have not been fully elucidated.

**Methods:**

To explore the effects of Dex on apoptosis, cell cycle, proliferation, senescence, osteogenic and adipogenic differentiation of hBMSCs at the various exposure times and concentrations, Hoechst 33342/PI staining, flow cytometry, crystal violet assay, β-galactosidase (β-GAL) activity assay, alizarin red S (ARS) staining assay, and Oil Red O (ORO) staining assay were performed. A microarray assay was used to identify differentially expressed lncRNAs and mRNAs in 10^− 6^ mol/L Dex-treated hBMSCs, and a bioinformatics analysis was conducted to further explore the role of these differentially expressed lncRNAs and mRNAs in the coding and noncoding (CNC) network. Furthermore, the microarray results were validated using quantitative real-time PCR (qRT-PCR) analysis.

**Results:**

Over the range of 10^−8^, 10^−7^, and 10^−6^ mol/L, Dex induced apoptosis, arrest of the cell cycle, inhibition of osteogenic differentiation, and promotion adipogenic differentiation of the hBMSCs in a dose-dependent manner. In addition, 10^−6^ mol/L Dex significantly induced apoptosis, suppressed proliferation, and increased the senescence of hBMSCs in a time-dependent manner. Interestingly, this time-dependent effect of Dex on the apoptosis of hBMSCs plateaued at the 7th day and decreased from the 8th day to the 10th day, while Dex treatment increased senescence of the hBMSCs on the 6th day. Furthermore, the microarray analysis identified a total of 137 differentially expressed mRNAs (90 upregulated and 47 downregulated) and 90 differentially expressed lncRNAs (61 upregulated and 29 downregulated) in hBMSCs after exposure to 10^−6^ mol/L Dex. The differentially expressed mRNAs and lncRNAs were associated with the regulation of cell apoptosis, proliferation, and cell cycle. Meanwhile, several signaling pathways involved in these processes, including the mTOR signaling pathway, Ras signaling pathway, HIF-1 signaling pathway, NF-kappa B signaling pathway, and TGF-beta signaling pathway, also were identified through the interaction net in the significant pathways (Path-Net) analysis. Furthermore, the CNC network further identified 78 core regulatory genes involved in the regulation of apoptosis. Additionally, qRT-PCR was used to confirm the identity of the key differentially expressed mRNAs and lncRNAs found to be closely associated with cell apoptosis to confirm the reliability of the microarray dataset.

**Conclusions:**

In summary, the effect of Dex on apoptosis, cell cycle, proliferation, and osteogenic differentiation and adipogenic differentiation of the hBMSCs depended on exposure time and concentration. Continuous exposure to 10^−6^ mol/L of Dex for 7 days may be a suitable protocol for inducing the apoptosis of hBMSCs. Under this protocol, differentially expressed lncRNAs and mRNAs associated with apoptosis, cell cycle, and proliferation were identified, providing a new research direction for further studies.

**Supplementary information:**

The online version contains supplementary material available at 10.1186/s13287-020-02040-8.

## Introduction

Mesenchymal stem cells (MSCs), which can be isolated from multiple tissues, including bone marrow, skeletal muscle tissue, adipose tissue, and umbilical cord blood [1], have the ability of self-renewal and differentiation into osteocytes, osteoblasts, adipocytes, chondrocytes, and other embryonic lineages [2]. Based on the criteria proposed by the International Society for Cellular Therapy (ISCT), isolated MSCs are not a homogenous population due to the inclusion of various cell types, such as stem cells, progenitor cells, fibroblasts, and other types of cells, which also known as multilineage-differentiating stress enduring (Muse) cells [1, 3]. Although Muse cells have the ability to cope with genotoxic stress by activating DNA repair systems, continuous DNA damage caused by various chemical and physical stimuli can lead to abnormal apoptosis, senescence, cell cycle arrest, and proliferation inhibition of human bone marrow mesenchymal stem cells (BMSCs), leading to a significant impact on bone metabolism and may act as a trigger for various skeletal disorders [2–4].

Dexamethasone (Dex) is one of the most commonly used glucocorticoid (GC) drugs. Long-term use of Dex is limited by several adverse effects including, bone loss, low bone mass, risk of fragility fracture, and osteonecrosis [5]. Notably, many reports have demonstrated the time-dependent and dose-dependent variability in Dex-induced responses of MSCs [6, 7]. For example, a short and low dosage of Dex treatment of MSCs stimulated osteogenesis [8, 9], whereas long-term exposure to a high dosage (10^−6^ mol/L) induced apoptosis and inhibited the proliferation of the BMSCs [6, 7], due to its probable mechanism of action in osteoporosis (OP) and steroid-induced osteonecrosis of the femoral head (SONFH). However, to date, the exact mechanisms of action of Dex on BMSCs have only been poorly defined. This process not only involves apoptosis, cell cycle, proliferation, and senescence of the BMSCs, but also involves the regulation of gene transcription and various signaling pathways [10–14].

Long noncoding RNAs (lncRNAs) are a class of non-protein-coding genes that are more than 200 nucleotides in length [15]. Emerging evidence has suggested that lncRNAs participate in a wide variety of cellular processes, including apoptosis, proliferation, migration, and the differentiation of BMSCs [16, 17]. Functionally, lncRNAs regulate gene expression via interfering with DNA, mRNA, or proteins [15].

In this study, we explored the effects of exposure to Dex on apoptosis, cell cycle, proliferation, senescence, and osteogenic and adipogenic differentiation of hBMSCs for various exposure times and concentrations. It was found that continuous exposure to 10^−6^ mol/L of Dex for 7 days may be a suitable protocol for inducing the apoptosis of hBMSCs. Based on this finding, we used microarray and bioinformatics assays to identify differentially expressed lncRNAs and mRNAs in a high dosage of Dex-treated hBMSCs, to reveal the potential role of these differentially expressed lncRNAs and mRNAs by predicting the interactions between coding and noncoding genes. Furthermore, the microarray results were validated using quantitative real-time PCR (qRT-PCR).

## Material and methods

### Isolation and culture of the hBMSCs

This study was approved by the Ethics Committee of the Affiliated Hospital of Qingdao University (Qingdao, China). Bone marrow samples were collected from three patients with fracture of the femoral neck (age 45, 47, and 52 years old) during total hip arthroplasty (THA) surgery conducted at the Department of Orthopedics at the Qingdao University Affiliated Hospital. All donors provided written informed consent. Cells were isolated and purified from the bone marrow tissue using density gradient centrifugation, as previously described [18], and were then cultured in Dulbecco’s modified Eagle’s medium (DMEM, Solarbio, Beijing, China) containing 10% (v/v) fetal bovine serum (FBS, Gibco, Thermo Scientific, Australia) and 100 units/mL penicillin-streptomycin (Solarbio, Beijing, China) in a 5% CO_2_ atmosphere at 37 °C. The cells were passaged at a 1:2 ratio after they had reached 90% confluency. Cells at passage 3 were used in all experiments.

### Phenotypes of the hBMSCs

Flow cytometric analysis was used to assess the expressions of the surface markers on the hBMSCs using an Apogee A50-MICRO flow cytometer (Apogee, UK), as described below. In brief, cells were digested using trypsin, centrifuged, and resuspended in cold phosphate-buffered saline (PBS) containing 1% FBS. After the concentration was adjusted to 1 × 10^6^ cells/mL, the cell suspension was incubated with the following antibodies: 20 μL of anti-CD34-PE, 20 μL of anti-CD45-PE, 5 μL of anti-CD73-FITC, and 2 μL of CD90-FITC (BD Biosciences, USA), respectively, for 30 min in dark at 37 °C. After the cells were washed three times with cold PBS, 100 μL of the single-cell suspension was used for the flow cytometric analysis. Untreated cells were used as a negative control.

### Assessment of the morphology of the apoptotic cells

Cells at passage 3 were grown in 24-well plates and were treated with 10^−6^ mol/L Dex for 10 days. From day 1 to day 10, a chromatin dye Hoechst 33342/PI Kit (Solarbio, Beijing, China) was used to assess the morphology of the apoptotic cells, following the manufacturer’s instructions. Apoptotic cells, which showed morphological characteristics, such as chromatic agglutination, karyopyknosis, and nuclear fragmentation, were identified and counted under a fluorescent microscope. Necrotic cells emitted a red hyperfluorescence and a blue hyperfluorescence. In addition, Hoechst 33342/PI staining assay of the hBMSCs treated with various concentrations of Dex (10^−8^ mol/L, 10^−7^ mol/L, and 10^−6^ mol/L) was also performed for 7 days. The hBMSCs were treated with the solvent of Dex as the control. The experiment was performed in triplicate.

### Flow cytometric analysis to determine apoptosis

Cells at passage 3 were grown in 6-well plates and treated with 10^−6^ mol/L Dex for 10 days. From day 1 to day 10, a Annexin V-PE/7-AAD Kit (BD Biosciences, USA) was used to analyze the percentage of apoptotic cells using a Apogee A50-MICRO flow cytometer (Apogee, UK), as recommended by the manufacturer, and at least 10^4^ cells were analyzed in each sample. In addition, flow cytometric analysis of the hBMSCs treated with various concentrations of Dex (10^−8^ mol/L, 10^−7^ mol/L, and 10^−6^ mol/L) was also performed for 7 days, as mentioned above. The hBMSCs were treated with the solvent of Dex as the control. The experiment was performed in triplicate.

### Flow cytometric analysis of the cell cycle

The effects of various concentrations of Dex (10^−8^ mol/L, 10^−7^ mol/L, and 10^−6^ mol/L) on the cell cycle of the hBMSCs were evaluated using a Cell Cycle Detection Kit (Solarbio, Beijing, China), as recommended by the manufacturer. In brief, cells at passage 3 were grown in 6-well plates and treated with Dex. Then, the hBMSCs at the logarithmic growth phase were collected and used to determine the cell cycle using flow cytometry (Apogee A50-MICRO, UK). FlowJo version 10 (BD, USA) was used to analyze the data, and at least 10^4^ cells were analyzed in each sample. The hBMSCs were treated with the solvent of Dex as the control. The experiment was performed in triplicate.

### Cell proliferation assay

The effect of Dex on hBMSC proliferation was evaluated using crystal violet assay. In brief, cells at passage 3 were grown in 96-well plates at an initial density of 2 × 10^4^ cells/well and treated with 10^−6^ mol/L Dex for 7 days. Then, the cells were fixed using 4% paraformaldehyde for 10 min and stained with 0.25% crystal violet solution for 30 min. After washing three times, the cells were observed under an inverted phase-contrast microscope and then 10% acetic acid was added to dissolve the crystal violet. Subsequently, crystal violet content was quantified at 570 nm using a microplate reader (Tecan, Austria). The experiment was performed in triplicate.

### β-Galactosidase (β-GAL) activity assay

The effect of Dex on the senescence of the hBMSCs was evaluated using β-GAL activity assay. In brief, cells at passage 3 were grown in 6-well plates and treated with 10^−6^ mol/L Dex for 10 days. From day 1 to day 10, cells were collected and counted, and then β-galactosidase (β-GAL) activity was detected using a β-GAL activity Kit (Solarbio, Beijing, China), as described by the manufacturer. Absorbance at 400 nm was measured using a microplate reader (Tecan, Austria), and β-GAL activity (nmol/h/10^4^ cell) was calculated, as recommended by the manufacturer.

### Osteogenic and adipogenic differentiation of the hBMSCs

For osteogenic differentiation, after 60% confluency was reached, the hBMSCs were cultured in an osteogenic induction medium that contained complete medium supplemented with 50 μg/mL ascorbic acid (Solarbio, Beijing, China), and 10 mmol/L β-glycerophosphate (Solarbio, Beijing, China), with Dex (10^−8^, 10^−7^, and 10^−6^ mol/L) for 14 days [19]. The osteogenic induction medium was replaced every 2 days. The hBMSCs were treated with the solvent of Dex as the control.

For adipogenic differentiation, after 80% confluency was reached, the hBMSCs were cultured in an adipogenic induction medium that contained complete medium supplemented with 500 μmol/L isobutylmethylxanthine (Solarbio, Beijing, China), 100 μmol/L indomethacin (Solarbio, Beijing, China), and 10 μg/mL insulin (Solarbio, Beijing, China), with or without Dex (10^−8^, 10^−7^, 10^−6^ mol/L) for 4 days. Then, the adipogenic induction medium was changed to a maintenance medium that contained the complete medium supplemented with 10 μg/mL insulin and 5 μmol/L pioglitazone (Solarbio, Beijing, China), with or without Dex (10^−8^, 10^−7^, and 10^−6^ mol/L) for 10 days [19]. The medium was replaced every 2 days. The hBMSCs were treated with the solvent of Dex as the control.

### Alizarin red S (ARS) staining assay

The osteogenic differentiation of hBMSCs after treatment with the osteogenic induction medium was evaluated using ARS staining assay. In brief, the cells were fixed using 4% paraformaldehyde for 20 min and stained with 0.2% ARS solution 15 min. After washing three times, mineralization nodule formation in the cells was observed under an inverted phase-contrast microscope. The experiment was performed and analyzed in triplicate.

### Oil Red O (ORO) staining assay

The adipogenic differentiation of the hBMSCs after treatment with the adipogenic induction medium was assessed using ORO staining assay. In brief, the cells were fixed using 4% paraformaldehyde for 20 min and stained with ORO solution (Solarbio, Beijing, China) for 15 min. After washing three times, lipid droplet formation in the cells was observed under an inverted phase-contrast microscope. The experiment was performed and analyzed in triplicate.

### Western blotting analysis

The protein expression levels of the osteogenic markers (BSPII and Runx-2) and adipogenic markers (PPAR-γ and CEBP-α) were detected using western blotting analysis. Briefly, cells at passage 3 were grown in 6-well plates and treated with various concentrations of Dex (10^−8^ mol/L, 10^−7^ mol/L, and 10^−6^ mol/L) for 48 h. The hBMSCs were treated with the solvent of Dex as the control. Total proteins from the hBMSCs were extracted using a RIPA Lysis Buffer (Solarbio, Beijing, China) and a protein loading buffer (EpiZyme, Shanghai, China) heated at 95 °C after total protein density was determined using a BCA Protein Detection Kit (Solarbio, Beijing, China). The protein samples were separated using SDS-PAGE and electrotransferred onto a PVDF membrane (Merck-Millipore, France). Then, the membrane was blocked, and in order incubated with primary antibodies and secondary antibodies. After visualization using ECL-PLUS reagents (Merck-Millipore, France), the target bands were scanned using a BioSpectrum Imaging System (UVP, USA). The integrated density was used to quantify the results of the western blotting analysis using ImageJ software (version 1.52u), and the results were normalized using GAPDH.

The primary antibodies, including rabbit anti-human BSPII, Runx-2, PPAR-γ, and CEBP-α antibodies, were purchased from Cell Signaling Technologies (Danvers, USA). The primary antibody for rabbit anti-human GAPDH and all secondary antibodies were purchased from Elabscience (Shanghai, China). All antibodies were diluted in the antibody dilution (Boster Biological Technology, Shanghai, China) at an appropriate ratio specified by the manufacturer.

### Microarray assays

The hBMSCs were cultured in complete DMEM containing 10% FBS and 100 units/mL of penicillin-streptomycin along with 10^−6^ mol/L Dex (Dex-induced group, Dex) or with the solvent of Dex (control group, Control). After treatment for 7 days, total RNA from was extracted from the hBMSCs in the two groups using a RNAiso plus kit (Takara Bio Inc., Kusatsu, Japan), following the manufacturer’s instructions. The purity and concentration of the RNAs were assessed at OD260/280 using a spectrophotometer (NanoDrop ND-1000). Total RNA was reverse-transcribed into cDNA, which was labeled using a fluorescent dye (Cy5 and Cy3-dCTP) and hybridized with the Agilent human lncRNA+mRNA Array V4.0 designed with four identical arrays per slide (4 × 180 K format). The microarrays were washed and then scanned using a G2565CA Microarray Scanner (Agilent). The lncRNA+mRNA array data were analyzed for data summarization, normalization, and quality control using GeneSpring software V13.0 (Agilent). A fold change of ≥ 2.0 or ≤ 2.0 and a *P* value (*t* test) of < 0.05 were used as threshold values to select differentially expressed lncRNAs and mRNAs. The experiment was performed and data were analyzed in triplicate.

### Bioinformatics analysis

DAVID Bioinformatics Resources 6.8 (https://david.ncifcrf.gov/) was used to conduct Gene ontology (GO) and pathway enrichment analyses. GO enrichment analysis was performed to identify the functions of the differentially expressed genes between the two groups, including the biological processes, cellular components, and molecular functions involved. Pathway enrichment analysis was performed using Reactome, KEGG, PID, PANTHER, BioCarta, and BioCyc. Furthermore, the coding-non-coding gene co-expression (CNC) network was constructed based on the correlation analysis between mRNA and lncRNA expression (Pearson correlation coefficients > 0.99 or ≤ 0.99). A *P* value of < 0.05 was considered to indicate statistical significance.

### Quantitative real-time PCR (qRT-PCR)

Differentially expressed mRNAs and lncRNAs were selected at random to confirm the results of the microarray assays using qRT-PCR. This was conducted in addition to the confirmation of the mRNA expression levels of osteogenic markers (BSPII and Runx-2) and adipogenic markers (PPAR-γ and CEBP-α). In brief, total RNA was obtained from the hBMSCs in the two groups using the RNAiso plus kit (Takara Bio Inc., Kusatsu, Japan), and then reverse transcribed into cDNA using a PrimeScript RT reagent kit (Takara Bio Inc., Kusatsu, Japan). qRT-PCR was performed on a Roche LightCycler 480 Detection System (Roche, Switzerland), using the SYBR Premix Ex Taq II kit (Takara Bio Inc., Kusatsu, Japan), following the manufacturer’s instructions. All forward and reverse primers used for the genes were provided by the Ribobio Corporation (Guangzhou, China) and are listed in Table 1. The relative expression level of each gene was evaluated using the 2^-△△Ct^ method and normalized to GAPDH. The experiment was performed and data were analyzed in triplicate.

**Table 1:** The sequences of primers for qRT-PCR

Gene	Primer sequence (5′-3′)
BSPII	F: CAGAGGAGGCAAGCGTCACT R: CTGTCTGGGTGCCAACACTG
Runx-2	F: TGTCATGGCGGGTAACGAT R: AAGACGGTTATGGTCAAGGTGAA
PPAR-γ	F: CCTATTGACCCAGAAAGCGATT R: CATTACGGAGAGATCCACGGA
CEBP-α	F: CTTCAGCCCGTACCTGGAG R: GGAGAGGAAGTCGTGGTGC
IFNG-AS1	F: GACAACATGGTACATGTGGCTAG R: CCTCGGTTGCTTTGATTACACA
STXBP5-AS1	F: GAGATTTAGGTGGGGACGCTGC R: AGGGACTTGCCTTGTCGCTGAT
MIR210HG	F: GCTTGGTAGAGTGTCACGCC R: CATCTGACCGAGCCAGTTTG
ZFHX4-AS1	F: GCGCTCAGAAGTTTACAAGG R: CTCTAGCTGAGTCTTCTGCT
CDK1	F: AGCCGGGATCTACCATACCC R: TCGAGAGCAAATCCAAGCCA
CIT	F: ATATGGAGCGCGGAATCCTTT R: TCAGCTATGGTGTCGGAATACT
GINS4	F: TCAAGCCTGTAATCCCAGCA R: GTTCAAGCGATTCTCCTGCC
BCL2-L11	F: GCATCATCGCGGTATTCGGT R: TCTGGTAGCAAAAGGGCCAG
CDH11	F: CCGTACAGTTGGTGGAAGGG R: ACGTGTACTGGGCTCTCTCT
SAMHD1	F: AGTATGTGGGTGAGACGCAG R: GGAAGAGATTCATAGTCCTCCCTT
GAPDH	F: GGTCACCAGGGCTGCTTTTA R: GGATCTCGCTCCTGGAAGATG

*qRT-PCR* quantitative real-time PCR, *BSPII* bone sialoprotein II, *Runx-2* runt-related transcription factor-2, *PPAR-γ* peroxisome proliferator-activated receptor-γ, *CEBP-α* CCAAT/enhancer-binding protein-α, *CIT* citron rho-interacting serine/threonine kinase; *CDK1* cyclin-dependent kinase 1; *GINS4* GINS complex subunit 4; *BCL2-like 11* B cell lymphoma 2-Like 11; *CDH11* cadherin 11; *SAMHD1* SAM domain and HD domain 1; *F* forward; *R* reverse

### Statistical analysis

SPSS 19.0 software (IBM, Armonk, NY, USA) was used to conduct all statistical analyses. One-way analysis of variances (ANOVA) was performed to compare data between more than three groups, and the unpaired *t* test was performed to compare data of two groups. All data are presented as mean ± SD, and a *P* value of < 0.05 was considered to indicate statistical significance. All charts were constructed using GraphPad Prism 8 software (GraphPad, CA, USA).

## Results

### Phenotypes of the hBMSCs

**Fig. 1:**
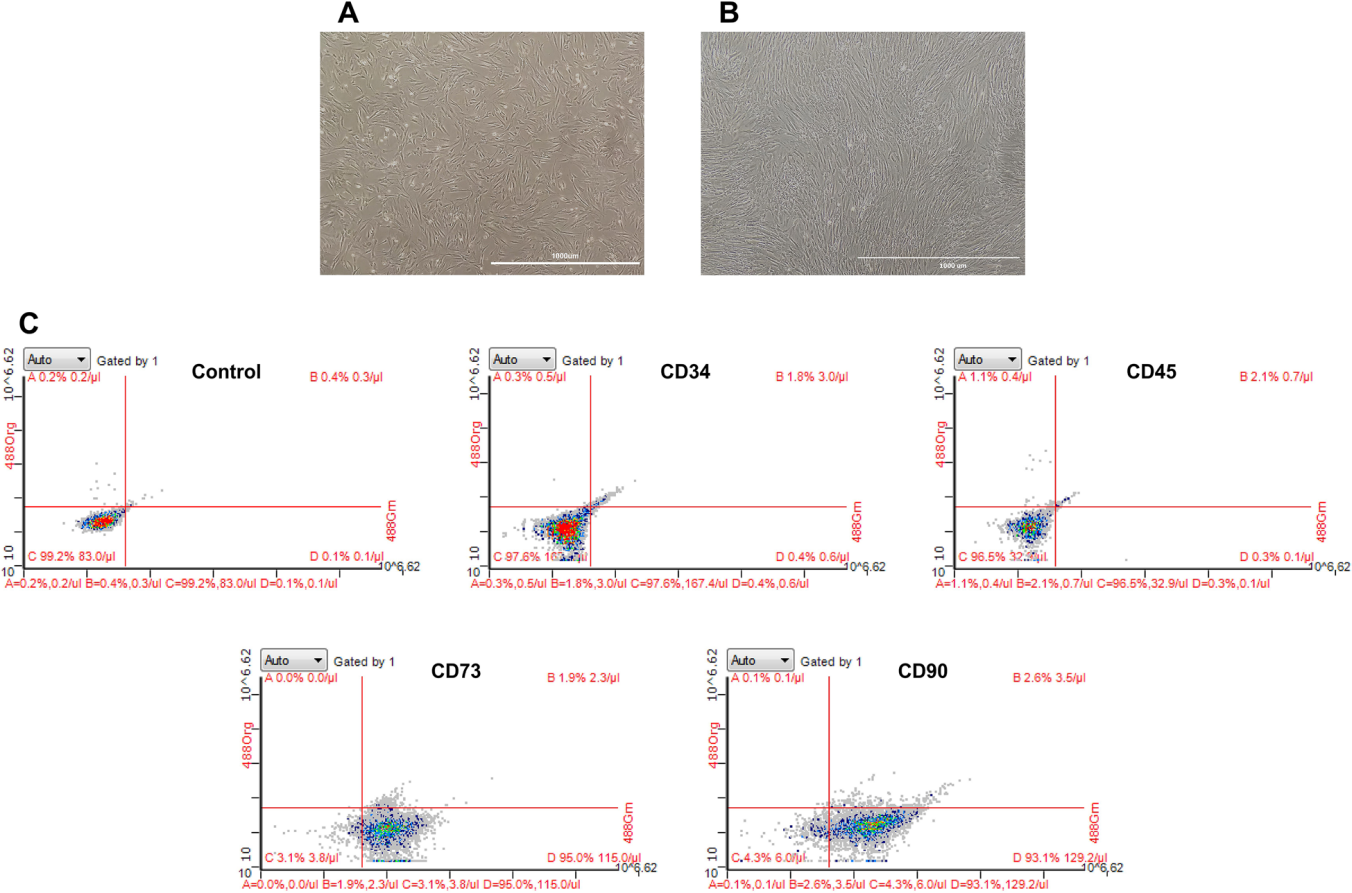
Identification of hBMSCs. Representative images showing the morphology of hBMSCs under an inverted phase contrast microscope: **a** cells at passage 3 at 40% confluency (scale bar = 1000 um) and **b** cells at passage 3 at 100% confluency (scale bar = 1000 um). **c** Phenotypic analysis of the hBMSCs using flow cytometry (CD34, CD45, CD73, and CD90). hBMSCs, human bone marrow mesenchymal stem cells; ALP, alkaline phosphatase

After two to three passages, the cultured cells homogeneously exhibited fibroblast-like characteristics and spindle-shaped morphology (Fig. 1a, b). Phenotyping of the hBMSCs using flow cytometry showed that these cells were positive for CD73 (95%), and CD90 (93.1%) (two cell surface markers of marrow-derived stem cells), but negative for CD34 (97.6%) and CD45 (96.5%) (two specific cell surface markers of hematopoietic cells) (Fig. 1c).

### Dex-induced apoptosis of hBMSCs depended on exposure time and concentration

**Fig. 2:**
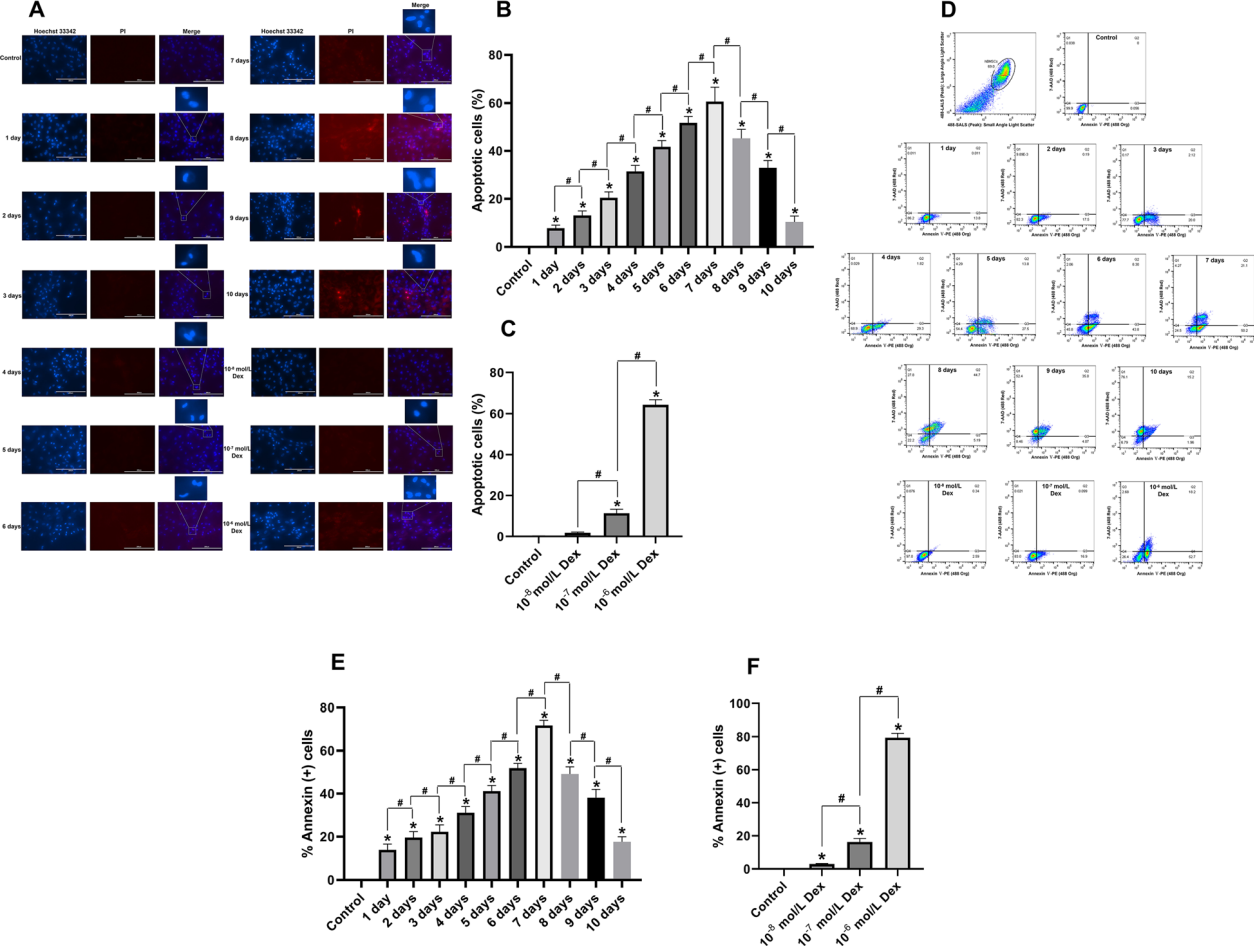
Dex-induced apoptosis of hBMSCs. Hoechst 33342/PI staining showing **a** apoptotic cells investigated under a fluorescence microscope (scale bar = 200 um), and **b** percentage of apoptotic cells after treatment with 10^−6^ mol/L Dex from day 1 to day 10 calculated through cell counting, and **c** percentage of apoptotic cells after treatment with various concentrations of Dex (10^−8^ mol/L, 10^−7^ mol/L, and 10^−6^ mol/L) for 7 days. Flow cytometry analysis showing **d** apoptotic cells after treatment with 10^−6^ mol/L Dex from day 1 to day 10 evaluated using Annexin V-PE and 7-AAD staining: Q1 shows necrotic cells, Q2 shows late apoptotic cells, Q3 shows normal cells, while Q4 shows early apoptotic cells. **e** percentage of Annexin^+^ cells after treatment with 10^−6^ mol/L Dex from day 1 to day 10. **f** percentage of Annexin^+^ cells after treatment with various concentrations of Dex (10^−8^ mol/L, 10^−7^ mol/L, and 10^−6^ mol/L) for 7 days. Note: all results are presented as mean ± standard deviation of three independent experiments. *P < 0.01 compared with the control group. hBMSCs, human bone marrow mesenchymal stem cells; Dex, dexamethasone; PI, propidium iodide; Q, quadrant

The effects of different exposure times and concentrations of Dex on the proliferation of hBMSCs were evaluated using Hoechst 33342/PI staining and flow cytometry analysis, respectively. Hoechst 33342/PI staining demonstrated that apoptotic cells grew significantly in number in a time-dependent manner after continuous exposure to 10^−6^ mol/L Dex from day 1 day to day 10. Remarkably, the effects plateaued on the 7th day and increased from day 8 to day 10 along with the increase in necrotic cells (Fig. 2a, b). Moreover, Dex significantly increased the number of apoptotic cells in a dose-dependent manner over the range of 10^−8^, 10^−7^, and 10^−6^ mol/L, after continuous treatment for 7 days (Fig. 2a, c). Likewise, flow cytometry analysis of Annexin V-PE/7-AAD double-staining showed a similar time and dose-dependent effect upon hBMSC exposure to Dex (Fig. 2d–f). Based on the above results, continuous treatment with 10^−6^ mol/L of Dex for 7 days may be a suitable protocol for inducing the apoptosis of hBMSCs, which is consistent with the results of previous studies [20].

### Effect of Dex on the cell cycle of hBMSCs depended on the exposure concentration

**Fig. 3:**
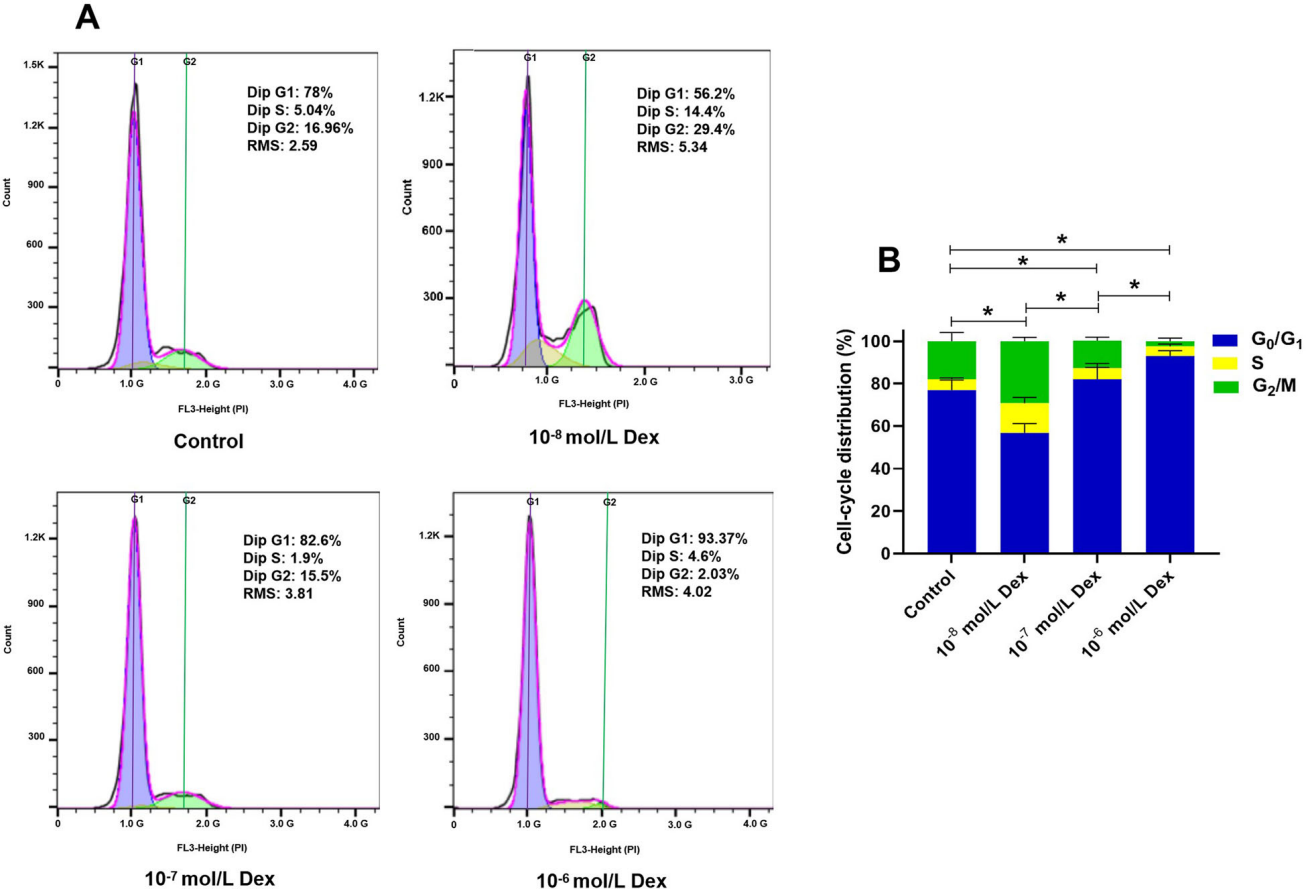
Effect of Dex on the cell cycle of the hBMSCs. **a** The results of the cell cycle distribution of the hBMSCs after exposure to various concentrations of Dex (10^−8^ mol/L, 10^−7^ mol/L, and 10^−6^ mol/L) were analyzed using FlowJo software. **b** The histogram showed the percentage of cells at each phase. Note: all results are presented as mean ± standard deviation of three independent experiments. *P < 0.01 between the two groups at the G0/G1 phase. hBMSCs, human bone marrow mesenchymal stem cells; Dex, dexamethasone

The cell cycle distribution of the hBMSCs after exposure to various concentrations of Dex (10^−8^, 10^−7^, 10^−6^ mol/L) was evaluated using flow cytometric analysis. The results showed that 10^−8^ mol/L Dex significantly increased the percentage of hBMSCs at the S and G2/M phase, but 10^−7^ mol/L Dex, especially 10^−6^ mol/L, arrested the cell cycle of the hBMSCs at the G0/G1 phase, compared with the control (Fig. 3a, b). As a result, the effect of Dex on the cell cycle of the hBMSCs depended on exposure concentration. Specifically, a low dosage of Dex had a positive effect, while a high dosage of Dex produced a negative effect on the cell cycle of the hBMSCs.

### 10^−6^ mol/L Dex inhibited the proliferation and induced the senescence of hBMSCs in a time-dependent manner

**Fig. 4:**
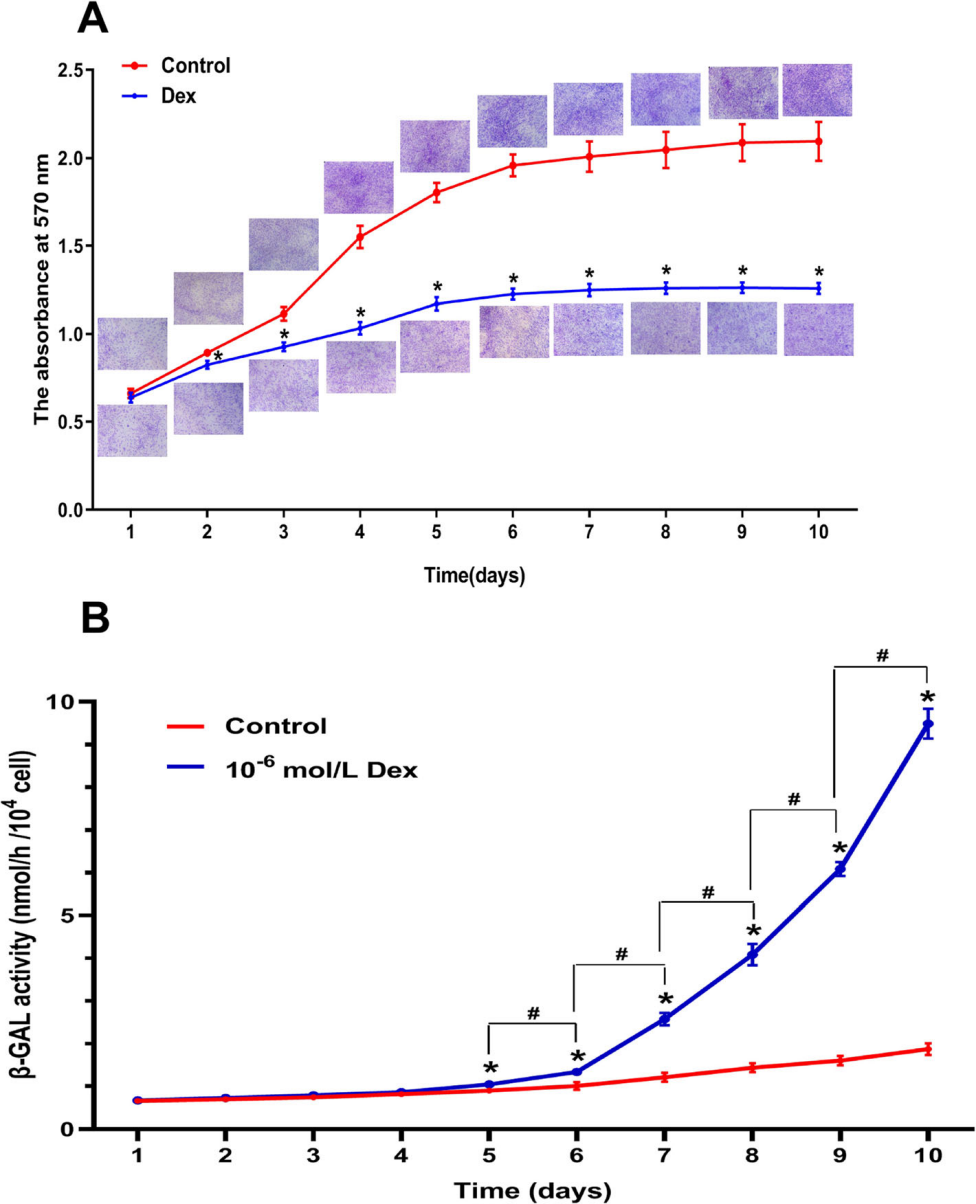
Effect of 10^−6^ mol/L Dex on the proliferation and senescence of the hBMSCs. **a** hBMSC proliferation viability after exposure to 10^−6^ mol/L Dex from day 1 to day 10 was determined using crystal violet assay. **b** Senescence of the hBMSCs after exposure to 10^−6^ mol/L Dex from day 1 to day 10 was evaluated using β-GAL activity assay. Note: all results are presented as mean ± standard deviation of three independent experiments. *P < 0.01 compared with the control group; ^#^*P* < 0.01 between the two groups. hBMSCs, human bone marrow mesenchymal stem cells; Dex, dexamethasone; β-GAL, β-galactosidase

The proliferation of hBMSCs after exposure to 10^−6^ mol/L Dex from day 1 to day 10 was evaluated using crystal violet assay. As shown in Fig. 4a, the crystal violet activity of the hBMSCs decreased significantly on day 3 after exposure to 10^−6^ mol/L Dex, compared with the control. Moreover, the crystal violet activity of the hBMSCs plateaued on day 9 in the Control, but plateaued on day 7th in the 10^−6^ mol/L Dex treatment group. Collectively, these results revealed that 10^−6^ mol/L Dex significantly inhibited the proliferation of the hBMSCs in a time-dependent manner.

In addition, the senescence of the hBMSCs exposure to 10^−6^ mol/L Dex from day 1 to day 10 was evaluated using β-GAL activity assay. As shown in Fig. 4b, 10^−6^ mol/L Dex significantly increased the β-GAL activity in the hBMSCs from day 5, compared with the control, and the effects became increasingly evident, revealing that 10^−6^ mol/L Dex could induce the senescence of hBMSCs in a time-dependent manner.

### Dex-induction of osteogenic and adipogenic differentiation of the hBMSCs depended on the concentration

**Fig. 5:**
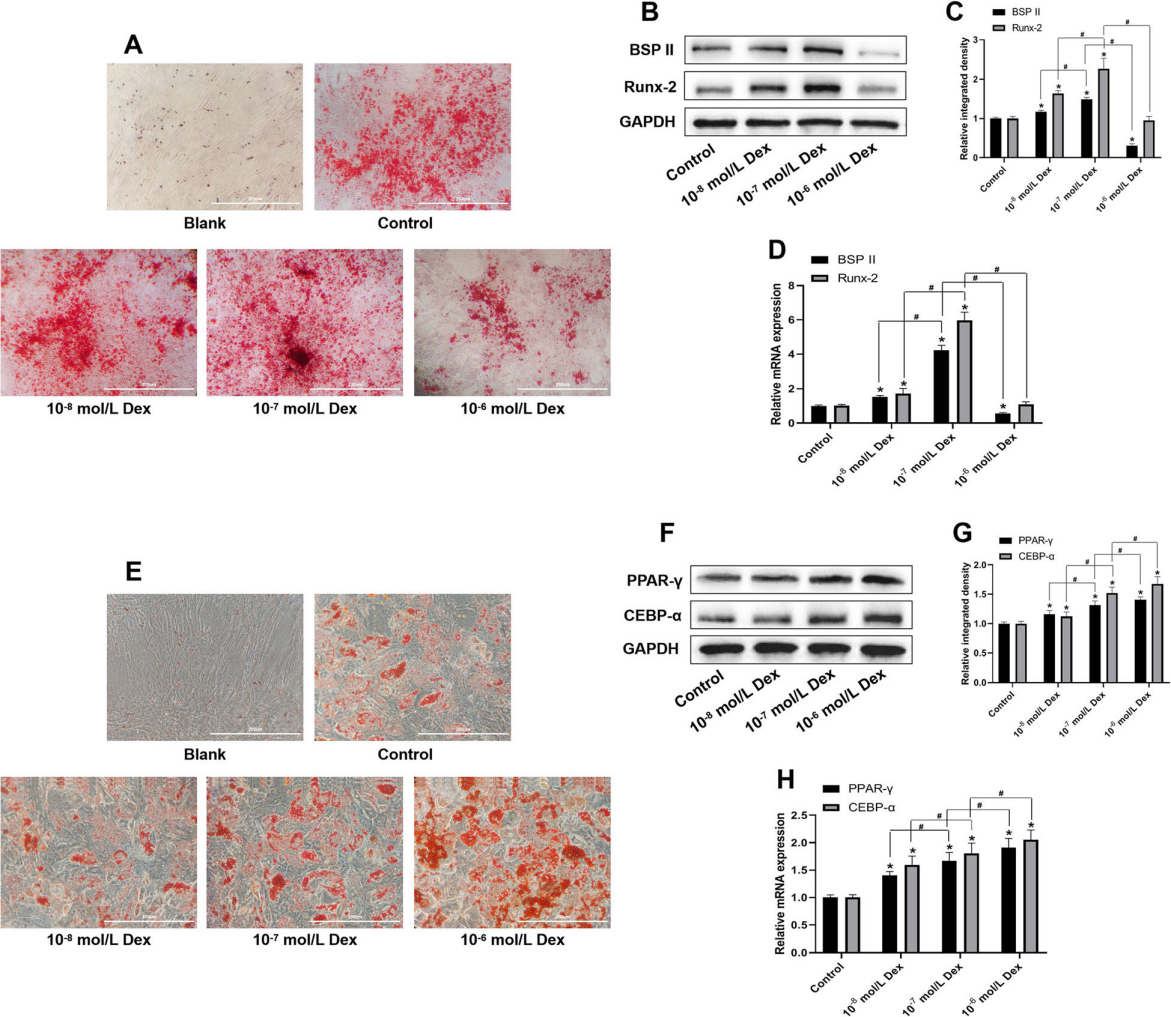
Effect of Dex on the osteogenic and adipogenic differentiation of the hBMSCs. **a** ARS staining of the hBMSCs after treatment with various concentrations of Dex (10^−8^ mol/L, 10^−7^ mol/L, and 10^−6^ mol/L) for 14 days. **b** The results of the western blotting analysis showing the protein expression levels of the osteogenic markers (BSPII and Runx-2) after treatment for 14 days, and **c** quantification of the integrated density of the target bands normalized to GAPDH levels. **d** The results of the qRT-PCR showing the mRNA expression of osteogenic markers (BSPII and Runx-2) after treatment for 14 days, and quantification through normalization to GAPDH levels. **e** ORO staining of the hBMSCs after treatment with various concentrations of Dex (10^−8^ mol/L, 10^−7^ mol/L, and 10^−6^ mol/L) for 14 days. **f** The results of the western blotting analysis showing the protein expression levels of the adipogenic markers (PPAR-γ and CEBP-α) after treatment for 14 days, and **g** quantification of the integrated density of the target bands after normalization to GAPDH levels. **h** The results of the qRT-PCR showing the mRNA expression levels of adipogenic markers (PPAR-γ and CEBP-α) after treatment for 14 days, and the quantification through normalization to GAPDH levels. Note: all results are presented as mean ± standard deviation of three independent experiments. *P < 0.01 compared with the control group; ^#^*P* < 0.01 between the two groups. hBMSCs, human bone marrow mesenchymal stem cells; Dex, dexamethasone; BSPII, bone sialoprotein II; Runx-2, runt-related transcription factor-2; PPAR-γ, peroxisome proliferator-activated receptor-γ; CEBP-α, CCAAT/enhancer-binding protein-α

The effect of various concentrations of Dex (10^−8^, 10^−7^, and 10^−6^ mol/L) on the osteogenic and adipogenic differentiation of the hBMSCs was evaluated using ARS and ORO staining assay, respectively. The results of ARS staining assay showed that the mineralization nodule formation and the expression of osteogenic markers (BSPII and Runx-2) in cells were significantly increased due to Dex at a concentration of 10^−8^ and 10^−7^ mol/L, but decreased significantly at a concentration of 10^−6^ mol/L (Fig. 5a–d), which revealed that 10^−7^ mol/L may be a suitable concentration to induce the osteogenic differentiation of the hBMSCs, while a high dosage of Dex (10^−7^ mol/L) produced the opposite effect. Interestingly, the results of ORO staining assay indicated that over the range of 10^−8^–10^−6^ mol/L, Dex significantly increased the lipid droplet formation and the expression of adipogenic markers (PPAR-γ and CEBP-α) in the cells in a dose-dependent manner (Fig. 5e–h), which revealed that 10^−6^ mol/L may be a suitable concentration to induce the adipogenic differentiation of hBMSCs.

### Differential expression profiles of mRNAs in Dex-induced apoptotic hBMSCs and bioinformatics analysis

**Fig. 6:**
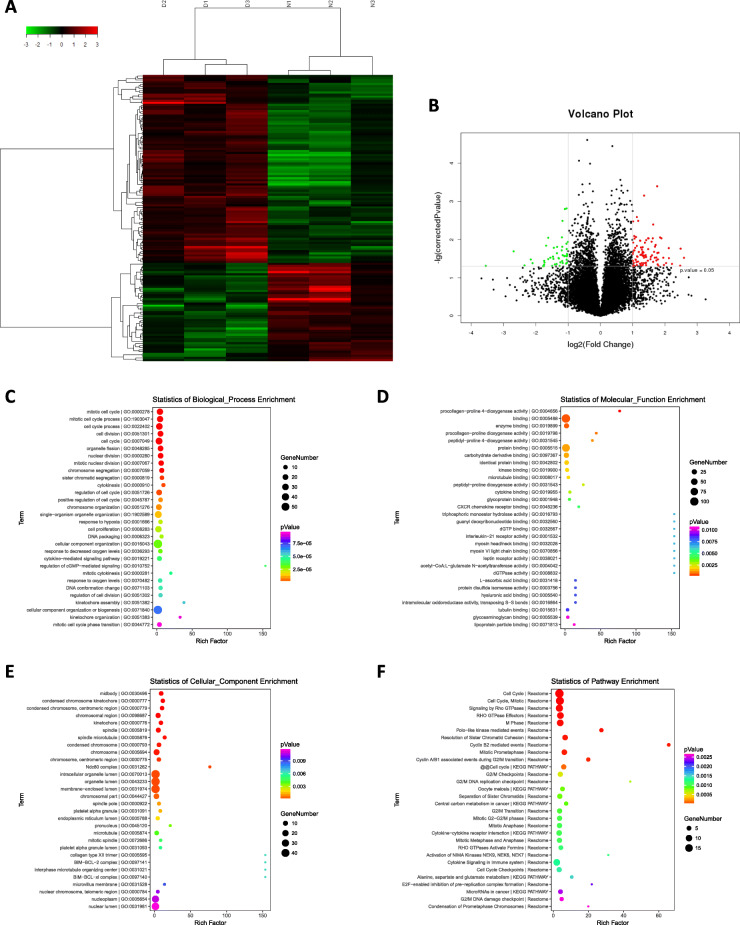
The differential expression profiles of the mRNAs in Dex-induced apoptotic hBMSCs and bioinformatics analysis. **a** A heat map of the distinct mRNAs based on the microarray assay. **b** A volcano plot of the distinct mRNAs based on thee microarray assay. Red dots represent upregulated mRNAs, and green dots represent downregulated mRNAs with statistical significance (fold change ≥ 2, *P* value < 0.05). Bubble maps of the GO enrichment analysis for **c** biological processes, **d** molecular functions, and **e** cellular components. **f** A bubble map of the pathway enrichment analysis showing the top 30 signaling pathways with significant changes. Notes: D, dexamethasone-induced group (*n* = 3); C, control group (*n* = 3); Dex, dexamethasone; hBMSCs, human bone marrow mesenchymal stem cells; GO, gene ontology; KEGG, Kyoto Encyclopedia of Genes and Genomes pathway analysis

The microarray identified a total of 137 differentially expressed mRNAs in Dex treated hBMSCs, compared with the control (FC ≥ 2, *P* value < 0.05), out of which 90 were upregulated, and 47 were downregulated (Fig. 6a, b, Supplemental file 1).

GO enrichment analysis revealed that the differentially expressed mRNAs were enriched in many biological processes, including regulation of the cell cycle, cell division, cell proliferation, cytokine-mediated signaling pathway, and cGMP-mediated signaling (*P* value < 0.05) (Fig. 6c), which are closely related to apoptosis [21, 22]. In the molecular function category, enrichment was seen in procollagen-proline 4-dioxygenase activity, enzyme binding, protein binding, carbohydrate derivative binding, kinase binding, and cytokine binding (*P* value < 0.05) (Fig. 6d). In the cellular component, they were enriched in midbody, condensed chromosome kinetochore, kinetochore, spindle, and intracellular organelle lumen, especially BIM-BCL-2 complex, which plays a significant role in promoting apoptosis [23] (*P* value < 0.05) (Fig. 6e).

Moreover, pathway enrichment analysis identified a total of 71 significantly differential signaling pathways (39 from Reactome, 13 from KEGG, 10 from PID, 3 from PANTHER, 4 from BioCarta, 2 from BioCyc). Among the top 30 signaling pathways, most of them were related to the regulation of apoptosis [24–26], such as cell cycle, signaling by Rho GTPases, polo-like kinase-mediated events, Cyclin B2 mediated events, and cytokine-cytokine receptor interaction (*P* value < 0.05) (Fig. 6f).

### Interaction net of significant pathways (Path-Net) of the differentially expressed mRNAs

**Fig. 7:**
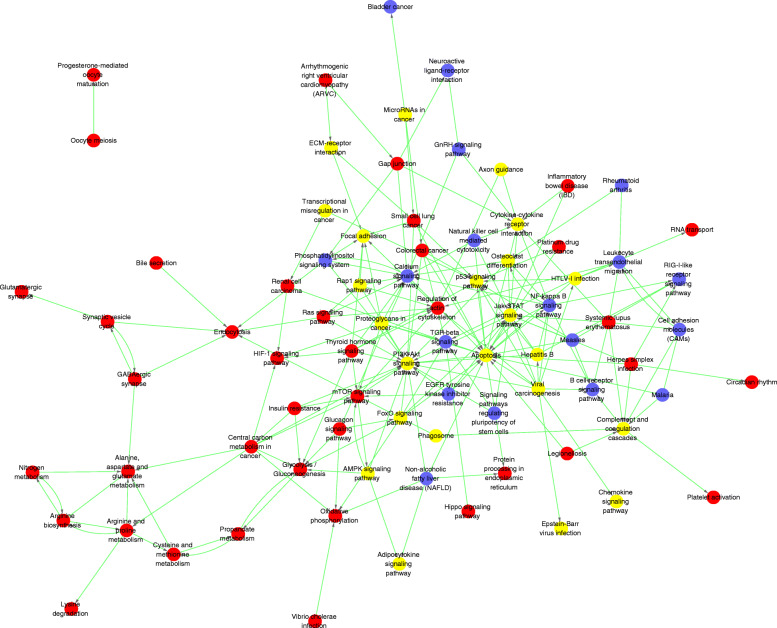
Interaction net of the significant pathways (Path-Net) of the differentially expressed mRNAs. Blue circle dots represent downregulated pathways, red circle dots represent upregulated pathways, while yellow circle dots represent upregulated and downregulated pathways. The lines show interactions between the pathways

The Path-Net of the differentially expressed mRNAs was constructed using the KEGG database to identify comprehensive interactions between significant pathways. Our results showed the upregulation of the signaling pathways mediated by mTOR, thyroid hormone, Ras, insulin resistance, HIF-1, and glucagon. In contrast, signaling pathways mediated through NF-kappa B, TGF-beta, and calcium, and pathways regulating the pluripotency of stem cells were downregulated (Fig. 7). Among these, mTOR, Ras, HIF-1, NF-kappa B, and TGF-beta signaling pathways have been confirmed to be associated with apoptosis [27–31].

### Differential expression profiles of lncRNAs in Dex-induced apoptotic hBMSCs

**Fig. 8:**
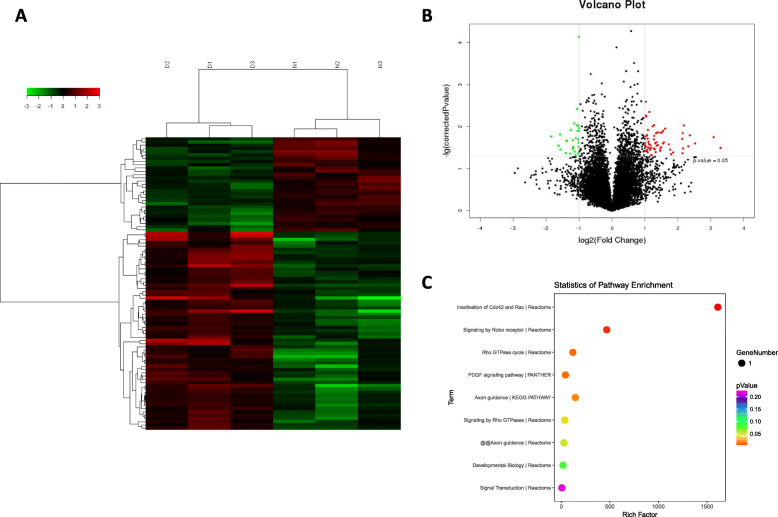
The differential expression profiles of the lncRNAs in Dex-induced apoptotic hBMSCs and bioinformatics analysis. **a** A heat map of the distinct lncRNAs based on the microarray assay. **b** A volcano plot of the distinct lncRNAs based on the microarray assay. Red dots represent upregulated lncRNAs, and green dots represent downregulated lncRNAs with statistical significance (fold change ≥ 2, *P* value < 0.05). **c** A bubble map of the pathway enrichment analysis. Notes: lncRNA, long noncoding RNA; D, dexamethasone-induced group (*n* = 3); C, control group (*n* = 3); Dex, dexamethasone; hBMSCs, human bone marrow mesenchymal stem cells; KEGG, Kyoto Encyclopedia of Genes and Genomes pathway analysis

A total of 90 differentially expressed lncRNAs were detected in Dex-induced apoptotic hBMSCs (FC ≥ 2, *P* value < 0.05). Among these, 61 were upregulated and 29 were downregulated, including 41 intergenic, 4 intronic, 5 divergent, 21 antisense, 17 uncategorized, and 2 previously unpublished lncRNAs (Fig. 8a, b, Supplemental file 2).

Furthermore, pathway enrichment analysis revealed a total of 6 significantly differential signaling pathways (4 from Reactome, 1 from KEGG, 1 from PANTHER), such as the inactivation of Cdc42 and Rac, signaling by Robo receptor, Rho GTPase cycle, and the PDGF signaling pathway (*P* value < 0.05) (Fig. 8c). Previous studies have reported that these signaling pathways are associated with the regulation of apoptosis, cell cycle, and cell proliferation [32–35].

### CNC network analysis of the key differentially expressed mRNAs and lncRNAs

**Fig. 9:**
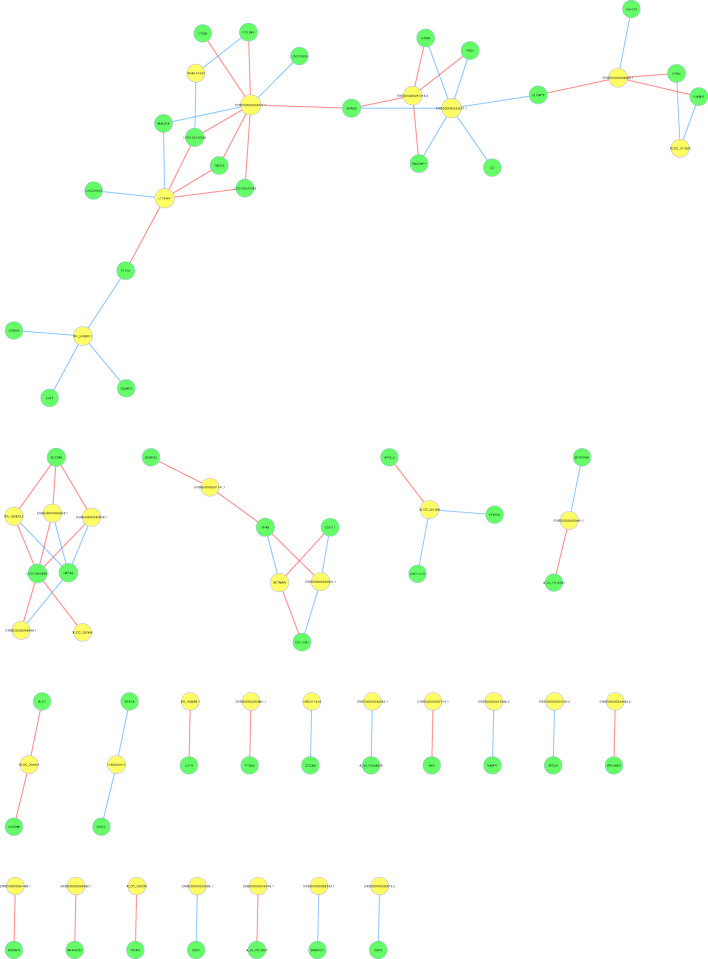
CNC network analysis of the key differentially expressed mRNAs and lncRNAs. Yellow circle dots represent lncRNAs, and green circle dots represent mRNAs. The size of the circle dot indicates the expression level of the gene. The lines show the interactions between the mRNAs and lncRNAs. Red lines indicate a positive correlation and blue lines indicate a negative correlation. Notes: CNC, coding and noncoding; lncRNA, long noncoding RNA

The CNC network was constructed using the correlation analysis, to evaluate interactions between differentially expressed lncRNAs and mRNAs. Our analysis identified a total of 78 core regulatory genes, including 47 mRNAs and 31 lncRNAs (Supplemental file 3). As shown in Fig. 9, 15 lncRNAs were correlated with one mRNA, whereas the others were correlated with two or more mRNAs. In particular, an interaction network was identified between the lncRNAs (XLOC_011523) and mRNAs (CDKN3, E2F7, and IQGAP3), which involved 21 mRNAs and 8 lncRNAs. Moreover, some key mRNAs, such as GINS4, CIT, CDK1, SAMHD1, and CDH11, were identified have been reported to be closely associated with the regulation of cell proliferation and apoptosis [36–40].

### Validation using qRT-PCR

**Fig. 10:**
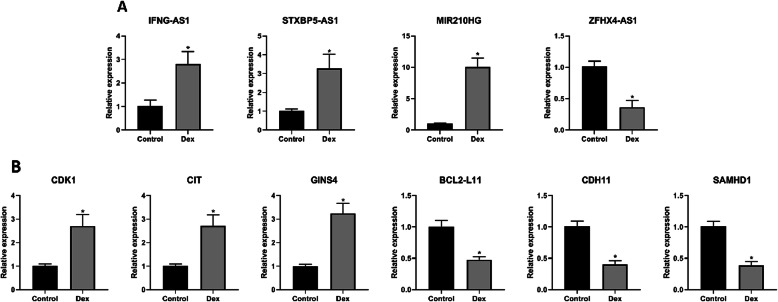
Confirmation of the differentially expressed mRNAs and lncRNAs using qRT-PCR. Note: all results are presented as mean ± standard deviation of three independent experiments. *P < 0.01 compared with the control group. Dex, dexamethasone-induced group; Control, control group; lncRNA, long noncoding RNA; qRT-PCR, quantitative real time polymerase chain reaction; CIT, citron rho-interacting serine/threonine kinase; CDK1, cyclin-dependent kinase 1; GINS4, GINS complex subunit 4; BCL2-like 11, B cell lymphoma 2-Like 11; CDH11, cadherin 11; SAMHD1, SAM domain and HD domain 1

We randomly selected 6 differentially expressed mRNAs (3 upregulated and 3 downregulated), and 4 differentially expressed lncRNA (3 upregulated and 1 downregulated) based on previous reports to confirm the reliability of the microarray data and facilitate downstream analysis (Table 2). Consistent with the microarray data, qRT-PCR validated the upregulation of the GINS complex subunit 4 (GINS4), citron rho-interacting serine/threonine kinase (CIT), and cyclin-dependent kinase 1 (CDK1), as well as the downregulation of BCL2-like 11 (BCL2-L11), SAM domain and HD domain 1 (SAMHD1), and cadherin 11 (CDH11) (Fig. 10a). Additionally, STXBP5-AS1, IFNG-AS1, and MIR210HG lncRNAs were upregulated, whereas ZFHX4-AS1 was downregulated (Fig. 10b).

**Table 2:** The details of mRNA and lncRNA selected for qRT-PCR confirmation

Gene name	Gene type	Regulation	Microarray (FC abs)	***P*** value	Class/cytoband
IFNG-AS1	lncRNA	Up	2.462	0.034624955	Antisense
STXBP5-AS1	lncRNA	Up	3.621	0.041374084	Intergenic
MIR210HG	lncRNA	Up	8.502	0.017894413	Intergenic
ZFHX4-AS1	lncRNA	Down	2.975	0.015331673	Intergenic
CDK1	mRNA	Up	3.105	0.038508828	hs|10q21.2
CIT	mRNA	Up	2.648	0.025473642	hs|12q24.23
GINS4	mRNA	Up	2.671	0.028762183	hs|8p11.21
BCL2-L11	mRNA	Down	2.257	0.003243677	hs|2q13
CDH11	mRNA	Down	3.203	0.014481736	hs|16q21
SAMHD1	mRNA	Down	2.440	0.031436465	hs|20q11.23

*qRT-PCR* quantitative real-time PCR, *CIT* citron rho-interacting serine/threonine kinase; *CDK1* cyclin-dependent kinase 1; *GINS4* GINS complex subunit 4; *BCL2-like 11* B cell lymphoma 2-Like 11; *CDH11* cadherin 11; *SAMHD1* SAM domain and HD domain 1; *FC abs* fold change absolute value; *lncRNA* long noncoding RNA

## Discussion

Dex-induced multilineage differentiation of MSCs into osteoblasts, adipocytes, skeletal muscle cells, and chondroblasts is accepted widely [2]. However, this diverse induction of MSCs as a result of Dex is dependent on concentration and exposure time [6–9]. In terms of the differentiation of the BMSCs, continuous treatment with 10^−7^ mol/L Dex facilitated osteogenic, adipogenic, and chondrogenic differentiation of BMSCs in the proper induction medium [41], while others have reported that transient treatment of Dex during the first week provided better stimulation for the osteogenesis of BMSCs, compared with constant exposure [42]. In this study, osteogenic differentiation of the hBMSCs was found to have significantly increased upon treatment with Dex at concentrations of 10^−8^ and especially at 10^−7^ mol/L, but the same significantly decreased at a concentration of 10^−6^ mol/L. Meanwhile, Dex significantly augmented the adipogenic differentiation of the hBMSCs via the upregulation of the expression of adipogenic markers (PPAR-γ and CEBP-α) in a dose-dependent manner over the range of 10^−8^–10^−6^ mol/L. Our results revealed that 10^−7^ mol/L may be the most suitable concentration for the induction of the osteogenic differentiation of the hBMSCs, but 10^−6^ mol/L may be suitable for inducing adipogenic differentiation.

Notably, shifting the focus from the multilineage differentiation of MSCs, recent studies have paid more attention to the pro-apoptotic effect of Dex. It has been reported that long-term exposure to a high dosage of Dex (10^−6^ mol/L) induced the apoptosis and inhibited the proliferation of BMSCs [6, 7]. Likewise, various concentrations of Dex produce diverse effects on the proliferation and apoptosis of MSCs. A low dose of Dex contributed to MSC proliferation, and prevented serum deprivation-induced apoptosis, whereas a high dose of Dex exacerbated the apoptosis of the MSCs [8]. Consistent with this report, our results also demonstrated that Dex induced the apoptosis of hBMSCs in a dose-dependent manner over the range of 10^−8^–10^−6^ mol/L, after continuous treatment for 7 days. Additionally, in this study, 10^−6^ mol/L Dex significantly induced apoptosis and suppressed the proliferation of hBMSCs in a time-dependent manner. However, it was interesting to note that the time-dependent effect of Dex on the apoptosis of the hBMSCs plateaued on day 7 and started to decrease on day 8, which is consistent with the results of a previous study [20]. Importantly, we found that 10^−6^ mol/L Dex induced the senescence of the hBMSCs in a time-dependent manner, but the increase in the senescence of the hBMSCs began on day 6. As far as we know, aging MSCs have a strong anti-apoptotic ability of decreasing the expression of apoptosis-related genes, compared with normal MSCs [43, 44]. In view of this, it is reasonable to presume that the plateau stage of 10^−6^ mol/L Dex-induced apoptosis of hBMSCs may be related with the senescence of the hBMSCs.

Furthermore, our previous study confirmed that arrest of the cell cycle was induced by 10^−6^ mol/L Dex [45]; therefore, we further explored whether this effect was related with the concentration of Dex in this study. Our data suggested that 10^−8^ mol/L Dex significantly increased the percentage of hBMSCs at the S and G2/M phases, but 10^−7^ mol/L Dex and especially 10^−6^ mol/L arrested the cell cycle of the hBMSCs at the G0/G1 phase, which indicated that different concentrations of Dex induce diverse effects on the cell cycle of the hBMSCs. Cell cycle arrest caused by a high dosage of Dex may act as a trigger to induce apoptosis, proliferation inhibition, and senescence of hBMSCs.

Nevertheless, the exact mechanism of action by which a high dosage of Dex affects MSCs remains equivocal. Emerging evidence has revealed that a large number of signaling pathways, such as the PI3K/Akt/mTOR [29], RAF-MEK-MAPK/ERK [46], NF-kB [29], and p53-dependent signaling pathways [47], have been implicated in apoptosis. These signaling pathways are regulated by a variety of transcripts, including coding and noncoding RNAs. LncRNAs are a type of noncoding RNA with a length of more than 200 nucleotides that have been reported to regulate apoptosis, proliferation, migration, and differentiation of MSCs by interfering with DNA, mRNA, or proteins [48–50]. However, crucial genes involved in the MSCs after exposure to a high dosage of Dex have got to be identified, despite reports that Dex can regulate the gene expression of the MSCs [51].

In this study, we utilized microarray analysis to identify differentially expressed lncRNAs and mRNAs in the hBMSCs after exposure to 10^−6^ mol/L Dex and identified 137 differentially expressed mRNAs (90 upregulated and 47 downregulated). The GO enrichment analysis demonstrated that these differentially expressed mRNAs were enriched in the regulation of the cell cycle, cell division, and cell proliferation-processes closely related to apoptosis. Moreover, pathway enrichment analysis identified a total of 71 significantly differential signaling pathways involved in the cell cycle, signaling by Rho GTPases, polo-like kinase-mediated events, Cyclin B2 mediated events, and cytokine-cytokine receptor interaction, which were closely related to the regulation of apoptosis [24–26]. Furthermore, Path-Net analysis highlighted that signaling pathways mediated by mTOR, Ras, HIF-1, NF-kappa B, and TGF-beta may play critical roles in Dex-induced apoptosis of hBMSC, which is consistent with the results of previous reports [27–31]. Notably, many signaling pathways indicated that the regulation of apoptosis proceeded through cross-talk.

Also, 90 differentially expressed lncRNAs (61 upregulated and 29 downregulated) were identified in our microarray assay. Pathway enrichment analysis identified a total of 6 significantly differential signaling pathways. Among them, inactivation of Cdc42 and Rac, signaling by the Robo receptor, Rho GTPase cycle, and the PDGF signaling pathway have been previously reported to be associated with the regulation of apoptosis. For example, both Cdc42 and Rac, subgroup members of Rho GTPases, suppressed apoptosis through the regulation of cell cycle progression [32, 34]. Moreover, the Robo receptor has also been reported to regulate various cellular processes, including cell proliferation, apoptosis, adhesion, and migration [33]. The PDGF family can be divided into four subtypes: PDGF-A, PDGF-B, PDGF-C, and PDGF-D. Among these subtypes, PDGF-D plays a key role in the regulation of proliferation, apoptosis, migration of cancer cells [35, 52], as well as the apoptosis of hepatic stellate cells [53].

The CNC network was constructed to investigate interactions between differentially expressed mRNAs and lncRNAs in Dex-induced apoptotic hBMSCs identified certain key mRNAs, such as SAMHD1, CDK1, GINS4, CDH11, and CIT, which were closely associated with regulation of cell proliferation and apoptosis. In addition, some important lncRNAs identified included ENSG00000233901.1, ENSG00000251018.2, ENSG00000255733.1, ENSG00000226605.1, ENSG00000233901.1, ENSG00000230921.1, and SETMAR. Most importantly, there was a significant correlation between these key transcripts. For instance, GINS4 was positively correlated with ENSG00000251018.2, but negatively with ENSG00000233901.1. CDH11 was positively correlated with SETMAR, and negatively correlated with ENSG00000230921.1. Additionally, SAMHD1, CDK1, and CIT were negatively correlated with ENSG00000255733.1, ENSG00000226605.1, and ENSG00000233901.1, respectively. Although the relationship between these lncRNAs was included in the CNC network and apoptosis has not been previously reported, they may regulate apoptosis by interacting with mRNAs, due to interactions between them.

Next, we selected 6 differentially expressed mRNAs and 4 differentially expressed lncRNAs, which have been reported in previous studies, to confirm the reliability of the microarray data and facilitate further analysis. Gene expression analysis using qRT-PCR confirmed that both GINS4, CIT, and CDK1 were upregulated, and that BCL2-L11, SAMHD1, and CDH11 were downregulated. Additionally, lncRNAs STXBP5-AS1, IFNG-AS1, and MIR210HG were upregulated, while ZFHX4-AS1 was downregulated. GINS4, also known as SLD5, plays an important role in the initial stages of DNA replication. Downregulation of GINS4 promoted cell cycle arrest, growth inhibition, and apoptosis in colorectal cancer cells [36]. Inactivation of CIT, a serine/threonine kinase, increased apoptosis, suppressed proliferation, and arrested the cell cycle via the regulation of Cyclophilin A in PDAC cells [37]. Similarly, inhibition of CDK1, a mitosis-promoting factor, promoted the apoptosis of cancer cells through G_2_/M arrest [38]. BCL2-L11, a member of the Bcl-2 family, performs a pro-apoptotic function in cardiomyocytes due to its interaction with Bcl-2 [54, 55]. SAMHD1, a mammalian dNTP hydrolase (dNTPase), increased apoptosis levels and decreased the level of proliferation of cancer cells by regulating the G_1_/G_0_ phase [39]. CDH11, which belongs to the cadherin family, induced apoptosis and inhibited cell proliferation by arresting the cell cycle at the G_0_/G_1_ phase in colorectal cancer cell lines [40].

The lncRNA IFNG-AS1 has been reported to inhibit apoptosis and promote proliferation, invasion, and migration of HP75 cells [56]. Likewise, lncRNA ZFHX4-AS1 repressed the apoptosis of breast cancer cells by regulating the Hippo signaling pathway [57]. In addition, the anti-apoptotic effects of MIR210HG in cancer cells promoted proliferation and invasion in cervical cancer [58]. On the other hand, STXBP5-AS1 promoted apoptosis, suppressed proliferation, and the invasion of MCF-7 cancer cells [59]. It can be stated that both mRNAs (GINS4, CIT, and CDK1) and lncRNAs (IFNG-AS1, ZFHX4-AS1, and MIR210HG) may be anti-apoptotic, whereas BCL2-L11, SAMHD1, and CDH11, as well as STXBP5-AS1, may also be pro-apoptotic in specific cell lines. It is interesting to note that the upregulation of STXBP5-AS1 (apoptosis activators) and the downregulation of ZFHX4-AS1 (apoptosis inhibitors) in Dex-induced hBMSCs were consistent in both the qRT-PCR and microarray analysis. Contrarily, the expressions of the other 8 genes were inconsistent with the pro-apoptotic effect induced by Dex on hBMSCs. To our knowledge, the regulation of apoptotic genes is cell-type dependent. For instance, previous studies have demonstrated that the lncRNA H19 inhibited MC apoptosis [49], but promoted apoptosis in cardiomyocytes and hippocampal neurons [60, 61]. Hence, given the cell-type specificity of Dex induced responses, the roles and underlying mechanisms of the genes identified in the present study need further elucidation.

## Conclusions

Collectively, the effect of Dex on apoptosis, cell cycle, proliferation, osteogenic differentiation, and adipogenic differentiation of hBMSCs depended on the exposure time and concentration. It is noteworthy that the time-dependent effect of Dex on the apoptosis of hBMSCs plateaued on day 7 and began to decrease from day 8, which may be related with the senescence of hBMSCs. As a result of this, continuous exposure to 10^−6^ mol/L of Dex for 7 days may be the most suitable protocol for the induction of the apoptosis of hBMSCs. Based on these results, we identified differentially expressed lncRNAs and mRNAs in the hBMSCs after exposure to 10^−6^ mol/L Dex using microarray analysis. Bioinformatics analysis methods, such as GO enrichment analysis, pathway enrichment analysis, and CNC network construction, further revealed the potential roles of these differentially expressed genes. This study provides evidence that further studies are required to reveal the exact action mechanisms of Dex on BMSCs.

## Supplementary Information

**Additional file 1.**

**Additional file 2.**

**Additional file 3.**

## Data Availability

The datasets included in this study are available from the corresponding author on reasonable request.

## Abbreviations

hBMSCs: Human bone marrow mesenchymal stem cells
Dex: Dexamethasone
GC: Glucocorticoid
OP: Osteoporosis
SONFH: Steroid-induced osteonecrosis of the femoral head
lncRNAs: Long noncoding RNAs
qRT-PCR: Quantitative real-time PCR
THA: Total hip arthroplasty
PBS: Phosphate-buffered saline
ALP: Alkaline phosphatase
GO: Gene ontology
CNC: Coding-non-coding gene
